# Improvement in Paretic Arm Reach-to-Grasp following Low Frequency Repetitive Transcranial Magnetic Stimulation Depends on Object Size: A Pilot Study

**DOI:** 10.1155/2015/498169

**Published:** 2015-11-17

**Authors:** Jarugool Tretriluxana, Shailesh Kantak, Suradej Tretriluxana, Allan D. Wu, Beth E. Fisher

**Affiliations:** ^1^Faculty of Physical Therapy, Mahidol University, Nakhon Pathom 73170, Thailand; ^2^Neuroplasticity and Motor Behavior Laboratory, Rehabilitation Research Institute, Elkins Park, PA 19027, USA; ^3^Faculty of Engineering, King Mongkut's Institute of Technology Ladkrabang, Bangkok 10520, Thailand; ^4^Department of Neurology, University of California Los Angeles, Los Angeles, CA 90095, USA; ^5^Division of Biokinesiology and Physical Therapy, University of Southern California, Los Angeles, CA 90089, USA; ^6^Department of Neurology, Keck School of Medicine, University of Southern California, Los Angeles, CA 90089, USA

## Abstract

*Introduction*. Low frequency repetitive transcranial magnetic stimulation (LF-rTMS) delivered to the nonlesioned hemisphere has been shown to improve limited function of the paretic upper extremity (UE) following stroke. The outcome measures have largely included clinical assessments with little investigation on changes in kinematics and coordination. To date, there is no study investigating how the effects of LF-rTMS are modulated by the sizes of an object to be grasped. *Objective*. To investigate the effect of LF-rTMS on kinematics and coordination of the paretic hand reach-to-grasp (RTG) for two object sizes in chronic stroke. *Methods*. Nine participants received two TMS conditions: real rTMS and sham rTMS conditions. Before and after the rTMS conditions, cortico-motor excitability (CE) of the nonlesioned hemisphere, RTG kinematics, and coordination was evaluated. Object sizes were 1.2 and 7.2 cm in diameter. *Results*. Compared to sham rTMS, real rTMS significantly reduced CE of the non-lesioned M1. While rTMS had no effect on RTG action for the larger object, real rTMS significantly improved movement time, aperture opening, and RTG coordination for the smaller object. *Conclusions*. LF-rTMS improves RTG action for only the smaller object in chronic stroke. The findings suggest a dissociation between effects of rTMS on M1 and task difficulty for this complex skill.

## 1. Introduction

Limited function of the paretic upper extremity (UE) is one of the most disabling consequences of stroke-related brain damage [1–5]. For example, there is an incorrect timing of components within movement sequences of an action [2]. Additionally, patients with either a mild or a moderate hemiparesis use different hand orientations for grasping and different patterns of trunk and upper limb compensation compared with nondisabled individuals [5]. In addition to impaired interjoint coordination [4], there are deficits in the regulation of interactive joint torques [3]. Clearly, reach-to-grasp (RTG) movement of the paretic limb is impaired after stroke. There has been research demonstrating improved UE function following low frequency repetitive transcranial magnetic stimulation (LF-rTMS) delivered to the nonlesioned hemisphere [6–13]. The improvements were reaction time and movement of Purdue Pegboard Test [6, 7], movement time of Nine-Hole Peg [9], pinch acceleration [10, 11], thumb abduction and other finger movements [8], kinematic of grasping [12], and RTG coordination [13]. The mechanism of this effect is considered to be restoration of balanced interhemispheric inhibition [14–17]. While the benefits following LF-rTMS are promising, outcome measures have largely been clinical assessments [6, 7, 9–11, 18, 19]. Very little is known regarding the effect of LF-rTMS on changes in kinematics and coordination of the UE [12, 13]. To date no study has investigated how the effect of LF-rTMS is modulated by task difficulty, that is, varying sizes of object to be grasped.

Reach-to-grasp (RTG) is a complex UE skill requiring coordination and precise scaling of hand aperture. It is one of the most important UE functions in activities of daily living and one in which individuals with stroke are particularly challenged. Importantly, the authors previously established differences between individuals with stroke and nondisabled adults in RTG kinematic measures of coordination and scaling. The measures were captured while the participants reached as fast as possible to pick up dowels of different sizes [20, 21]. Coordination was quantified using cross-correlation analysis between transport velocity and aperture size. The previous work established our unique measures that were valid and sensitive for capturing coordination changes in RTG as a result of stroke [21] as well as after intensive poststroke training [22].

In addition to coordination, a complex skill like RTG requires a visuomotor transformation for precise scaling of graspaperture to the spatial features of the object such as size. In a previous study by Nowak and colleagues [12], only kinematic variables of reach-to-grasp for one object size were studied. Based on the Fitts law [23], reaching to grasp the smaller object is more difficult than reaching to grasp the larger object. We recently reported part of the results on the effect of LF-rTMS for the smaller object on individuals with chronic stroke [13]. Therefore, the purpose of this study is to investigate the effect of LF-rTMS applied to the nonlesioned primary motor cortex (M1) on kinematics and coordination of paretic RTG for two object sizes, a smaller and a larger diameter object. If there is an improvement in RTG of the paretic limb for both objects, it would suggest a more general impact of LF-rTMS on a complex skill of the UE. Alternatively, if the effect is different for each object size, then it would suggest a dissociation between effects of LF-rTMS on M1 and task difficulty for this complex skill.

## 2. Methods

### 2.1. Participants

There were 9 participants who passed the inclusion and exclusion criteria. The inclusion criteria were as follows: (1) ischemic or hemorrhage unilateral stroke with onset more than 6 months, (2) age range 20–79 years, (3) right handedness evaluated by the Edinburgh Inventory Test, (4) mild to moderate impairment level of the UE Fugl-Meyer Assessment (FMA), (5) being able to understand and follow simple commands based on performance on the mini-mental state examination (MMSE) (cutoff > 23), (6) being able to reach and grasp a dowel at least once, (7) no unilateral visual neglect (star cancellation test > 44 points), (8) no ideomotor apraxia (imitation meaningless of gesture; error score ≤ 3), and (9) no motor aphasia, sensory aphasia, and global aphasia. The exclusion criteria were as follows: (1) positive screening for contraindication of rTMS which is confirmed by TMS screening questionnaire such as seizure and an intracranial metallic implant and (2) other neurological and musculoskeletal problems affecting arm, hand, or trunk which may interfere with task achievement.

All participants gave a written informed consent to participate in the experimental protocol approved by the Institutional Review Board of the University of Southern California.

### 2.2. Experimental Design

This study was a prospective cohort within-subject design. Patients participated in two sessions separated by at least 5 days. At each session, they performed the behavioral (RTG) task at baseline. This was followed by either real rTMS or sham rTMS condition. The order of sham and real rTMS was counterbalanced across participants. Five participants received real rTMS first while four participants received sham TMS during the first session. Following rTMS application, the participants repeated the behavioral task.

#### 2.2.1. Reach-to-Grasp (RTG) Task and Procedure

The behavioral task was to reach forward from a designated start position, grasp a prepositioned cylindrical dowel with the paretic hand, and lift it off the table as soon as indicated by a visual cue and as quickly as possible using the thumb and index finger. Each participant sat in a straight back chair with the torso secured to the chair back using a cross-shaped belt to minimize trunk motion. The participant's paretic hand rested on a hand switch, and the thumb and index fingertips were positioned in opposition. The vision of the hand was occluded at the start position. A light emitting diode (LED) located 25 cm above the table surface positioned behind the target cylindrical dowel provided the visual cue to begin each trial. Prior to each trial, the cylindrical dowel was positioned on an object switch located 30 cm from the start switch. Two cylindrical dowels, each measuring 10 cm in height, but of two diameters, were used. The smaller dowel measured 1.2 cm in diameter, while the larger dowel was 7.2 cm in diameter. For 5 participants, the small object was presented first for 10 trials, followed by the large object for 10 trials. For the other four participants, the large object was presented first in a 10-trial block, followed by 10 trials of the small object.

Reach-to-grasp kinematics data were acquired using the Motion Monitor system (Innovative Sports Training Inc., Chicago, IL), an electromagnetic motion system with 6 degrees of freedom sensors (Ascension Technologies). Three sensors were attached with tape to the paretic UE, one was on the forearm proximal to the styloid process of the radius, and the other two were on the nail bed of the thumb and index finger. Position data were captured at 120 Hz. The LED and hand and object lift switches were interfaced with the Motion Monitor through a timer and signaled the following 3 events: LED onset, hand lift onset, and movement termination (object lift switch).

#### 2.2.2. Magnetic Stimulation Procedures

Single pulse TMS was used to measure motor cortical excitability of the nonlesioned hemisphere. Stimulation was delivered with 70 mm figure of eight coils attached to Magstim Rapid2 magnetic stimulator and evoked responses were measured from the Extensor Digitorum Communis (EDC) muscle on the nonparetic side. First, the “hot-spot” was identified. It was the optimal scalp position for consistently eliciting the largest motor evoked potential (MEP) from the nonlesioned primary motor cortex (M1) representational area of EDC. The coil was held tangentially to the scalp with the coil-handle pointing posteriorly away from the midline at an angle of 45° [24, 25]. Next, resting motor threshold (MT) was determined by systematically decreasing the stimulus intensity over the hot-spot. For every participant, the experimenter started stimulation at 70% of the maximum stimulator output and modified the intensity based on the evoked responses. The stimulation intensity was reduced systematically until the motor threshold was determined. Motor threshold is defined as the lowest intensity level required to induce MEP peak-to-peak amplitude of at least 50 *μ*V, in 5 out of 10 consecutive trials [26]. Following determination of MT, 1 Hz rTMS was applied for 20 min (1200 pulses) at 90% of MT to the nonlesioned hemisphere EDC hot-spot as the real rTMS condition. Using these parameters, the authors previously demonstrated that LF-rTMS downregulated the motor cortical excitability as evidenced by a reduction in the size of the MEP amplitude after rTMS [17]. In the sham rTMS condition, a sham coil was positioned similar to the real condition and the same TMS parameters were employed. By using this type of coil, TMS clicking noise was produced but no TMS pulse was delivered. To confirm the downregulating effect of real rTMS on motor cortical excitability compared to the sham condition, MEPs were recorded before and after both rTMS conditions by applying 10 single TMS pulses at 120% MT over the EDC hot-spot of the nonlesioned hemisphere.

### 2.3. EMG Recording

Surface EMG was recorded from the nonparetic EDC muscle with surface electrodes (Motion Lab Systems) placed in a tendon-belly arrangement over the bulk of the muscle. The EMG signal was filtered with a bandpass of 1–1000 Hz, amplified, and digitized at 2000 Hz. The data were graphically displayed and stored for offline analysis.

### 2.4. Dependent Measures and Data Analysis: Corticomotor Excitability

MEPs were analyzed offline using* DataWizard*, a MATLAB-based program [27]. Peak-to-peak amplitude was computed for each recorded MEP. Mean MEP amplitude of 10 trials was calculated before and after the rTMS procedure. A paired *t*-test was used to compare the pre-TMS MEP amplitude to post-TMS MEP amplitude.

#### 2.4.1. Reach-to-Grasp Actions

**Figure 1:**
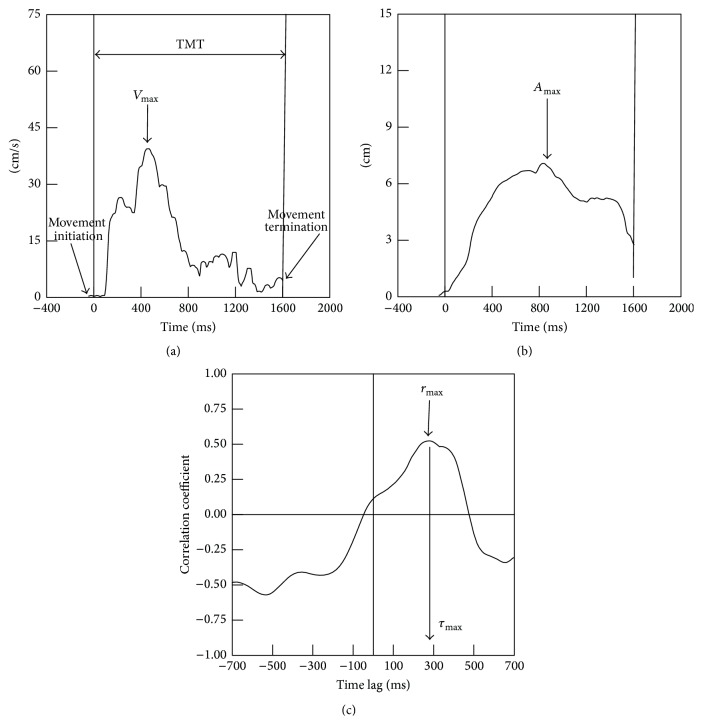
Key variables: (a) transport velocity with marked total movement time (TMT) and maximum transport velocity (*V*_max_), (b) grasp aperture with marked maximum grasp aperture (*A*_max_), and (c) transport-grasp coordination indicated by highest cross-correlation coefficient (upper arrow, *r*_max_) and associated time lag (lower arrow, *τ*_max_).

All kinematic data were analyzed by JT, who was blinded to the TMS conditions. The data were filtered using a zero-lag Butterworth low-pass filter with 20 Hz cutoff frequency. All kinematic and transport-grasp coordination variables were extracted for each trial using customized automatic computer routines written in MATLAB 7.5.0 (The MathWorks Inc., Natick, MA). Three-dimensional displacement was calculated from the wrist sensor position to derive tangential velocity of transport (Figure 1(a)) using a finite-difference technique [28]. Aperture was derived from the distance between the thumb and index finger sensors. Movement initiation was defined as the first bin of a continuous rise of at least 3 data points in transport velocity. Movement was terminated at the time of object lift-off.

The kinematic measures (Figures 1(a) and 1(b)) for the reach-to-grasp action included total movement time (time from movement initiation to movement termination), peak transport velocity (maximum value of the transport velocity trajectory), time of peak transport velocity (the occurrence of maximum transport velocity expressed as the percentage of total movement time), peak aperture (maximum value of the aperture trajectory), and time of peak aperture (the occurrence of maximum aperture expressed as the percentage of total movement time). Reach-to-grasp coordination (Figure 1(c)) was captured by the maximum cross-correlation coefficient between transport velocity and grasp aperture sizeand the associated time lag of cross-correlation. Our previous work demonstrated that a cross-correlation analysis provides a comprehensive way to capture both spatial and temporal coordination for RTG actions [20, 21]. Spatial coordination between the transport and grasp component was characterized using the cross-correlation coefficient (*r*) between transport velocity and grasp aperture. The emergent time lag from the cross-correlation analysis indicates the temporal coordination between the transport and grasp components. In our convention, a positive lag represents a lead of peak transport velocity over peak aperture. The higher (closer to 1) coefficient and shorter time lag (ranging from 80 to 125 ms) indicate stronger transport-grasp coordination [20]. *z* score (Fisher's *z*) transformed correlations were used for comparisons across participants. Transformed *z* scores were converted back to equivalent coefficients for reporting. A 2-TMS condition (real, sham) × 2-time (pre, post) ANOVA with repeated measures on the time was used to compare the effects of real rTMS to sham rTMS on the total movement time, peak transport velocity, peak aperture, time of peak transport velocity and time of peak aperture, cross-correlation coefficient, and associated time lag. Mauchly's test was used to test for sphericity assumption. If there was a violation, the Greenhouse-Geisser correction was applied. Wilcoxon Signed Ranks Test was used to compare baseline variables of the reach-to-grasp performance obtained from small and large objects.

To further explore if there was a relationship between changes in corticomotor excitability of the nonlesioned hemisphere and changes in the RTG action following rTMS, we determined the Pearson product (parametric) or Spearman (nonparametric) correlation (depending on normality) to see if it is different from zero. Significance level was set at *p* < 0.05. SPSS 16.0 (SPSS Inc., Chicago, IL) statistical software was used for all statistical analyses.

## 3. Results

Nine patients with hemiparesis (5M, 4F, mean age: 59 (6.8) years, age range: 48–69 years, right-hand dominant before stroke) participated in the study.  Table 1 summarizes the participant characteristics. The lesion locations were heterogeneous, originating from both anterior and posterior circulations. The mean time since stroke was 4.8 years (range from 7 months to 7 years). Their upper extremities were mildly impaired as evidenced by Motor Fugl-Meyer (FM) score of at least 45 out of 66. All participants presented with unilateral paresis. They did not show any suffering from their spasticity. They were able to successfully reach and grasp a dowel. Participants S2 and S5 who had the occipital lesion showed the signs of incoordinated actions, measured by the FM coordination item. All of them tolerated rTMS well with no adverse effects occurring or reported during or after the experiment.

### 3.1. Motor Cortical Excitability

The effect of 1 Hz rTMS over M1 on corticomotor excitability was examined by the change in the MEP amplitude between pre- and post-rTMS conditions (Table 1). While 1 Hz rTMS applied over the nonlesioned M1 significantly decreased MEP amplitude of the nonparetic EDC (paired *t*-test, *t*(8) = 3.648; *p* = 0.007), sham rTMS did not have a significant effect on MEP amplitude (paired *t*-test, *t*(8) = −0.581; *p* = 0.577). This finding confirmed 1 Hz rTMS as a valid tool for downregulating motor cortical excitability.

### 3.2. Reach-to-Grasp (RTG) Kinematics and Coordination

**Figure 2:**
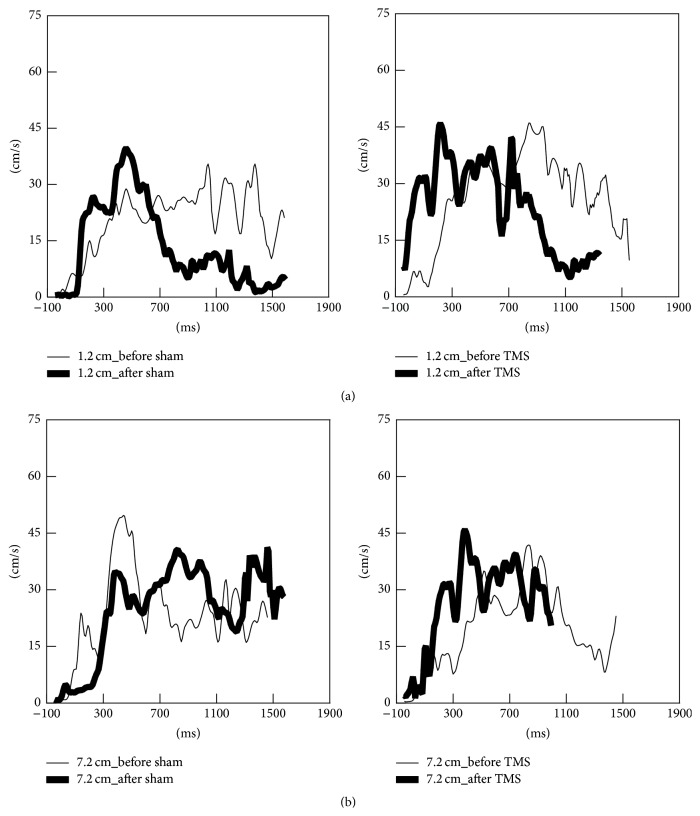
Representative data for transport velocity during reach-to-grasp actions to the small object (a) and the large object (b) following sham (left) and real rTMS conditions (right). The thin trajectories represent baseline performance; the thick trajectories represent performance after each TMS condition.

**Figure 3:**
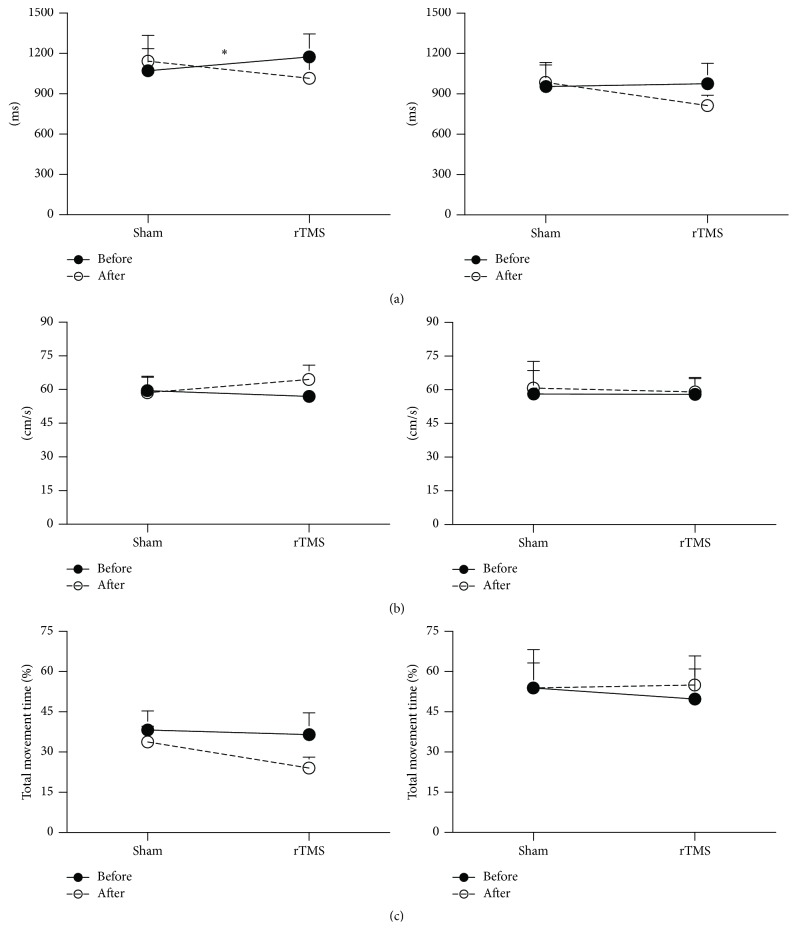
Kinematic parameters of the transport phase for the small object (left) and the large object (right) pre (—) and post (- - -) each TMS condition. (a) Effect on total movement time. (b) Effect on peak transport velocity. (c) Effect on time of peak transport velocity. *∗* indicates a significant interaction between the TMS condition and time. Error bar = SD.

**Figure 4:**
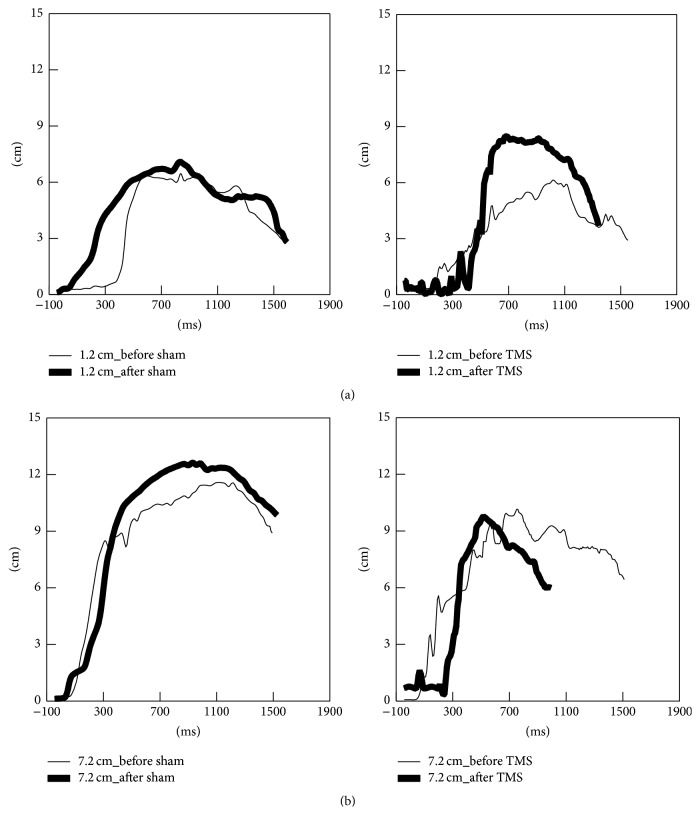
Representative data for grasp aperture during reach-to-grasp actions to the small object (a) and the large object (b) following sham (left) and real rTMS conditions (right). The thinner trajectories represent baseline performance; the thick trajectories represent performance after each TMS condition.

**Figure 5:**
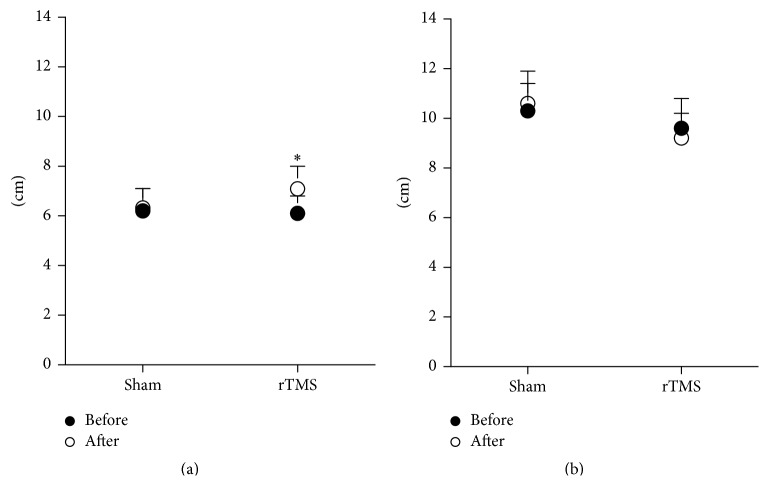
Grasp aperture for the small object (a) and the large object (b) pre (●) and post (○) each TMS condition. *∗* indicates a significant interaction between the TMS condition and time. Error bar = SD.

**Figure 6:**
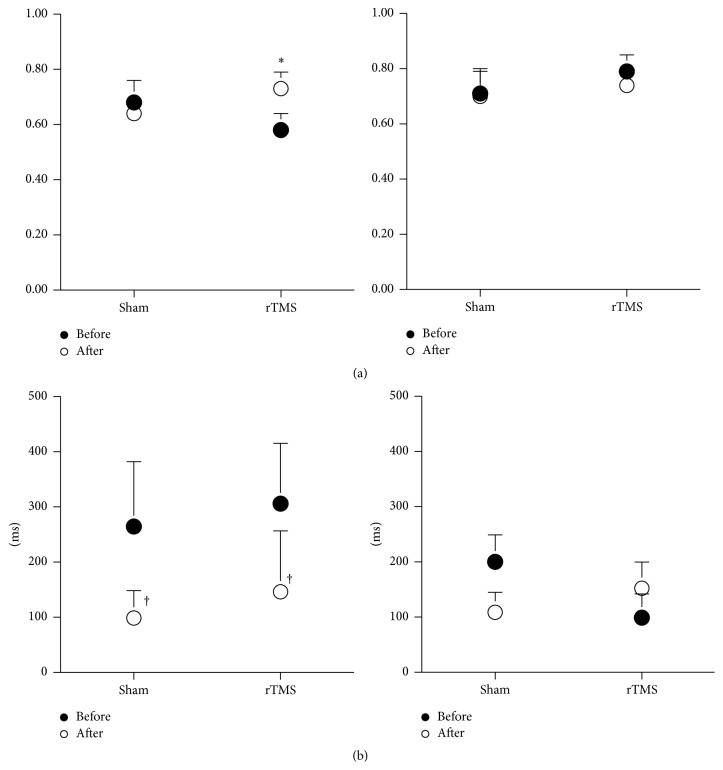
Reach-to-grasp coordination for the small object (left) and the large object (right) pre (●) and post (○) each TMS condition. (a) Highest cross-correlation coefficient. (b) Associated time lag. *∗* indicates a significant interaction between the TMS condition and time. † indicates a significant difference before compared to after TMS conditions.

All measures of RTG kinematics and RTG coordination of both large and small objects are shown in Figures 2–6.

#### 3.2.1. Baseline Measures

At baseline, collapsing across TMS condition, peak grasp aperture was significantly greater for the larger object compared to the smaller object (Wilcoxon Signed Ranks Test, *z*(8) = −2.599; *p* = 0.009) (Figure 5). Additionally, compared to the smaller object, the participants reached and grasped the larger object with a more coordinated pattern as evidenced by a greater maximum cross-correlation coefficient (Wilcoxon Signed Ranks Test, *z*(8) = −2.244; *p* = 0.025, Figure 6(a)) and shorter time lag (Wilcoxon Signed Ranks Test, *z*(8) = −2.497; *p* = 0.013, Figure 6(b)).

#### 3.2.2. Effect of rTMS on Transport Kinematics

Figure 2 illustrates representative subject data for the effect of sham and real rTMS on transport velocity for small and large objects. Following real rTMS, but not sham TMS, reach-to-grasp movements were faster for both small and large objects. However, the effects of LF-rTMS on improvements in movement time were statistically significant only for the small object (Figure 3). There was a significant reduction in the total movement time for the small object following real rTMS, but not sham rTMS (Figure 3(a), left column, TMS condition (real, sham) × time (pre, post) interaction, *F*(1,16) = 12.701, *p* = 0.004). Although a similar trend was observed for the larger object (Figure 3(a), right column), the effect was not statistically significant. Compared to sham, real rTMS did not significantly affect peak transport velocity and time of peak transport velocity for either the small or the large object (Figures 3(b) and 3(c)).

#### 3.2.3. Effect of rTMS on Grasp Kinematics

Figure 4 illustrates representative subject data showing the effect of sham and real rTMS on the temporal evolution of grasp aperture as the participant reached to grasp the small and large objects. The most prominent effect was an increase in the maximum grasp aperture for the small object following real LF-rTMS applied over the nonlesioned hemisphere. Figure 5 shows the data for maximum grasp aperture averaged across all participants. While there was little effect of real rTMS for the larger object RTG (Figure 5(b)), real rTMS significantly enhanced maximum grasp aperture while reaching to a smaller object compared to sham stimulation (Figure 5(a), TMS condition (real, sham) × time (pre, post) interaction, *F*(1,16) = 5.706, *p* = 0.034). There was no significant effect of rTMS on time to peak aperture for either object size.

#### 3.2.4. Effect on Coordination between the Transport and Grasp Components

Compared to sham stimulation, real rTMS did not significantly affect the spatial or temporal coordination between transport and grasp when participants reached to grasp the larger object (Figures 6(a) and 6(b), right columns). For the smaller object, spatial coordination (higher correlation coefficient) after rTMS compared to before rTMS improved with real rTMS, Figure 6(a), left column, TMS condition (real, sham) × time (pre, post) interaction, *F*(1,16) = 6.529, *p* = 0.034. Interestingly, participants also demonstrated a significant shortening of the time lag for the small object RTG action with both real and sham TMS conditions, time (pre, post) effect, *F*(1,16) = 7.419, *p* = 0.026.

#### 3.2.5. The Relationship between Improvement in RTG Kinematics and RTG Coordination and Changes in Corticomotor Excitability of the Nonlesioned Hemisphere

**Figure 7:**
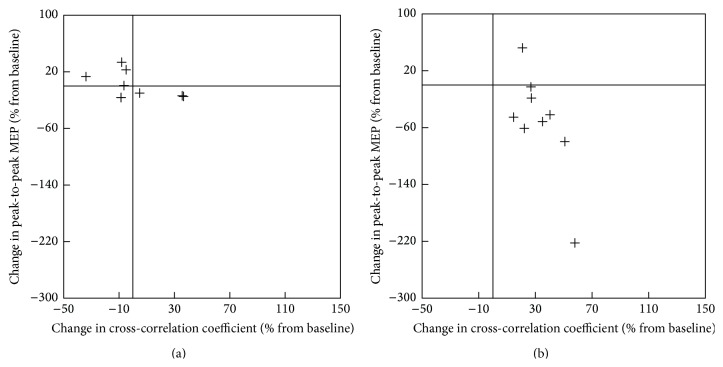
Scatter plots of the change in corticomotor excitability of the nonlesioned hemisphere and the change in cross-correlation coefficient of reach-to-grasp for the small object. (a) Sham TMS condition. (b) Real TMS condition.

While there was no significant correlation between changes in corticomotor excitability and RTG kinematics (*p* > 0.05), the decrease in corticomotor excitability of the nonlesioned hemisphere showed a strong correlation with an increase in cross-correlation coefficient of the RTG for the small object (*r* = −0.75, *p* < 0.05, Figure 7(b)).

## 4. Discussion

The authors observed paretic limb improvement in RTG kinematics and RTG coordination with the smaller object but not the larger object following LF-rTMS to M1 of the nonlesioned hemisphere. This result suggests a dissociative interaction between effects of rTMS on M1 and task difficulty for this complex skill.

### 4.1. Baseline Differences in RTG Kinematics and Coordination as a Function of Object Sizes

Compared to nondisabled adults, deficits in kinematics and coordination of reach-to-grasp components are observed following stroke. However, for every measurement used in this study, superior performance was observed when participants reached to grasp the large compared to the small dowel. Participants were faster and more coordinated, while achieving greater peak grasp aperture, supporting the notion that reaching to grasp the smaller object was more challenging than reaching to grasp the larger object as predicted by Fitts law [23].

One of the key invariant features that characterize normal RTG actions is the spatial and temporal coordination between the reach and grasp components. While Michaelson and colleagues [29] also reported slower movements in patients after stroke, they reported a relatively preserved coordination between reach and grasp components. They used the temporal delay between time to peak aperture and peak transport velocity as a measure of temporal coordination. In the current study, we used novel and more sensitive cross-correlation analysis to capture the coordination between reach and grasp [20, 21]. We demonstrate that spatial and temporal coordination between the reach and grasp components were impaired when participants reached to grasp the smaller object but not the larger object. The differences between our findings and those of Michaelson and colleagues could be attributed to a smaller object size in our study (1.2 cm diameter) compared to that used in the Michaelson study (3.3 mm diameter). Precision requirements to successfully reach and grasp smaller objects may put more demands on the patient for better coordination between body segments. Differences are also likely to arise from the outcome measure employed to study the coordination between reach and grasp components. Unlike using a single temporal event to characterize coordination, the cross-correlation analysis measures coordination of the entire spatiotemporal profile of transport velocity and grasp aperture by capturing every point in time of the RTG action.

### 4.2. Differences in RTG Kinematics and Coordination as a Function of Object Size following LF-rTMS

In the present study, the beneficial effects of rTMS over contralesional M1 on kinematics and coordination of RTG actions became more robustly evident for the small compared to the larger object. This suggests that task difficulty interacts with rTMS effects on RTG behavior. Object size influences task difficulty such that smaller objects require greater precision and thereby leads to greater engagement of the corticosubcortical networks compared to larger objects. Previous research has demonstrated more involvement of M1 contralateral to the performing hand during complex task execution [30–32]. After stroke, a higher IHI from the nonlesioned hemisphere may inhibit the engagement of the lesioned M1 during more complex movements of the paretic limb [15, 16]. Therefore, downregulation of nonlesioned M1 released the lesioned M1 from greater IHI, therefore allowing the lesioned M1 to be involved during performance of the more complex small object RTG action. Conversely, a more complex task likely provides a more sensitive measure for revealing behavioral effects following a neurophysiological intervention such as rTMS.

Participants in the current study were relatively mild in their impairments and demonstrated better coordination in reach-to-grasp with the larger compared to the smaller object. We think this is unlikely due to a ceiling effect for the larger object. This can be supported by (1) the fact that abnormalities for both objects were evident when compared to age-matched nondisabled adults performing the same task [20] and (2) improvements of RTG performance with a larger object in another treatment study [33]. Recently, we demonstrated that individuals with mild to moderate stroke were able to improve paretic arm movements after undergoing intensive task-specific motor training [33]. The limited improvement for the larger object following one session of LF-rTMS in this study may suggest the need for a greater number of TMS sessions or rTMS session in combination with motor training.

Interestingly, we observed a significant improvement in the time lag for the small object with sham rTMS. This improved time lag may be attributed to the effect of repeated performance of the task during the experiment. There is evidence to indicate that even short-term task practice with the paretic hand may improve some aspects of task performance. However, the fact that sham TMS did not improve any other measures offers evidence that real rTMS was more effective in improving the performance of RTG actions. Further, it is unlikely that the small number of repetitions as were performed in this study would contribute towards restoring normal interhemispheric balance, but it cannot be ruled out.

### 4.3. rTMS Applied over Nonlesioned M1 Improved the Kinematics and Coordination of RTG Actions

In this study, we provide further support for the notion that overactivity within the nonlesioned M1 is maladaptive and may impede performance of unimanual RTG actions of the paretic hand. Our findings of improved kinematics and coordination of paretic arm RTG are consistent with and extend previous findings. While Nowak and coworkers demonstrated improved kinematics of grasping following LF-rTMS applied to M1 [12], our data suggest that these improvements may be specific to task characteristics that, in our study, were determined by the object size. One potential mechanism underlying this effect is that the downregulation of excitability of the nonlesioned hemisphere following LF-rTMS serves to restore balanced interhemispheric inhibition [6, 8, 10, 34]. There is evidence that interhemispheric interactions are modulated by the kinematics of the movement [35]. It is possible that downregulation of the nonlesioned M1 decreased the IHI to the EDC representational area in ipsilesional M1, thereby improving kinematics of hand opening during RTG action. Disinhibition of the lesioned M1 with rTMS (over nonlesioned M1) may uncover its role within the neural network critical for RTG coordination of the paretic hand. Recent studies [36–38] support that M1 is a part of the neural network for high-level planning of the transformation between kinematics and dynamics [38]. It contributes to transformations between extrinsic representation and intrinsic representation of limb motor behavior. Therefore, at least a part of critical higher-level neuronal operations for RTG actions appears to reside in M1. These likely include coordination and the transformation of visual information (i.e., object characteristics and hand position) into an action. When “released” from abnormally high IHI, lesioned M1 is likely to be able to contribute more effectively as a part of the neural network to coordination of reach and grasp components.

An alternative but not mutually exclusive mechanism may be attributed to improved paretic hand opening observed following rTMS. There is evidence from an extensive body of behavioral research that change in one component (i.e., grasp) of RTG action alters the movement characteristics of the other component (i.e., reach) [39–42]. Therefore, improved hand opening following rTMS may have improved the coordination between reach and grasp components of the RTG action. This improved coordination with reduced movement time following rTMS, but not sham TMS, suggests a more efficient movement performance when nonlesioned M1 is suppressed.

### 4.4. Potential Limitations

Although we demonstrate that downregulation of the nonlesioned M1 was associated with improvement in the kinematics and coordination of RTG movements, we were unable to pinpoint the precise mechanism that led to those improvements. Additional assessment of interhemispheric inhibition using a paired-pulse technique would have allowed clearer insight into the mechanisms implementing the improvement in paretic hand motor behavior. In this study, we did not assess the participants' ability to distinguish between sham and real rTMS. This may potentially limit the blinding of the participant to the intervention. However, downregulation of MEP amplitude with real LF-rTMS but not sham indicates that real LF-rTMS was effective in reducing the excitability of the contralesional hemisphere.

Other limitations of the study include a small sample size as well as lack of lesion information for 4 participants. Additionally, the lesion information obtained revealed a heterogeneous sample. However, the within-subject design as well as the method of counterbalancing the order of TMS conditions allowed us to demonstrate valid and reliable findings. Our results showed a positive effect of LF-rTMS on RTG actions for the small object, but not the large object. With the small number of subjects, we may have been overoptimistic for this preliminary trial to detect the dissociation between effects of LF-rTMS on M1 and task difficulty for this skill. Larger sample size and more homogenous lesion are needed to guarantee the findings.

## 5. Conclusions

Here we demonstrate improved kinematics and coordination of reach-to-grasp for the smaller object in individuals with chronic stroke by decreasing the excitability of the nonlesioned M1. These results potentially extend support for the contralesional overexcitability hypothesis of persistent paretic arm deficits following chronic stroke. To our knowledge, this is the first study to investigate the coordination between reach and grasp using a functional RTG task, the object size constraints, and cross-correlation analysis after application of LF-rTMS.

## Figures and Tables

**Table 1:** Demographic information of 9 participants after stroke.

Subject ID	Age	Affected side	Lesion location	Time since stroke onset (years)	Upper extremity Fugl-Meyer score (out of 66)	Pre-TMS MEP (*μ*v) Nonlesioned hemisphere	Post-TMS MEP (*μ*v) Nonlesioned hemisphere
S1	53	R	L brainstem	7	59	832.4	403.9
S2	60	R	L occipital	4	52	1209.3	661.8
S3	58	R	L primary sensorimotor area, internal capsule, and caudate nucleus	7	55	667.8	260.1
S4	68	L	N/A	4.5	54	679.5	138.6
S5	62	L	R occipital	6.5	45	1206.8	700.6
S6	59	R	L pontine	3	55	885.1	274.7
S7	54	L	N/A	2.5	60	484.8	395.4
S8	48	L	N/A	3.6	50	293.9	447.2
S9	69	L	N/A	0.6	55	909.3	885.1
Mean (SD) counts	59.0 (6.8)	4 R/5 L		4.3 (2.2)	53.9 (4.5)	796.5 (304.3)	463.0 (240.9)
